# ERRATA

**DOI:** 10.1590/0034-7167.20227506e04

**Published:** 2022-05-27

No artigo “Percepção de mulheres na assistência ao parto e nascimento: obstáculos para a humanização”, com número de DOI: https://doi.org/10.1590/0034-7167-2021-0215 publicado no periódico Revista Brasileira de Enfermagem, 75(Suppl 2):e20210215, nas referências:

Onde se lia:

16. Tong A, Sainsbury P, Craig J. Consolidated criteria for reporting qualitative research (COREQ):a 32-item checklist for interviews and focus groups. Int J Qual Health Care. 2007;19(6):349-57. https://doi.org/10.1093/intqhc/mzm042

17. Fontanella BJB, Luchesi BM, Saidel MGB, Ricas J, Turato ER, Melo DG. Amostragem em pesquisas qualitativas: proposta de procedimentos para constatar saturação teórica. Cad Saúde Pública [Internet]. 2011[cited 2020 Feb 14];27(2):389-94. Available from: https://periodicos.fiocruz.br/pt-br/publicacao/15050

18. Gil AC. Métodos e técnicas de pesquisa social. 7º ed. São Paulo: Atlas. 2019. 248 p.

Lê-se:

16. Gil AC. Métodos e técnicas de pesquisa social. 7º ed. São Paulo: Atlas. 2019. 248 p.

17. Tong A, Sainsbury P, Craig J. Consolidated criteria for reporting qualitative research (COREQ):a 32-item checklist for interviews and focus groups. Int J Qual Health Care. 2007;19(6):349-57. https://doi.org/10.1093/intqhc/mzm042

18. Fontanella BJB, Luchesi BM, Saidel MGB, Ricas J, Turato ER, Melo DG. Amostragem em pesquisas qualitativas: proposta de procedimentos para constatar saturação teórica. Cad Saúde Pública [Internet]. 2011[cited 2020 Feb 14];27(2):389-94. Available from: https://periodicos.fiocruz.br/pt-br/publicacao/15050

